# The Main Determinants for Suicidal Ideation in a Romanian Cohort of Multiple Sclerosis Patients

**DOI:** 10.1155/2020/2594702

**Published:** 2020-01-21

**Authors:** Andreea Romaniuc, Rodica Bălaşa, Nicoleta Ştirbu, Smaranda Maier, Sebastian Andone, Zoltan Bajko, Laura Bărcuţean, Septimiu Voidăzan, Anca Moţăţăianu

**Affiliations:** ^1^Neurology Clinic I, Emergency Clinical County Hospital, Targu Mures, Romania; ^2^Doctoral School (I.O.S.U.D.) of the University of Medicine, Pharmacy, Science and Technology of Târgu Mureș, Romania; ^3^Department of Neurology, University of Medicine, Pharmacy, Science and Technology of Targu Mures, Romania; ^4^Municipal Hospital, Sighisoara, Romania; ^5^Department of Epidemiology, University of Medicine, Pharmacy, Science and Technology of Targu Mures, Romania

## Abstract

**Objective:**

To determine the prevalence of suicidal concerns (SC) in a large multiple sclerosis (MS) patient group and to assess the major determinants that are implicated in their occurrence.

**Methods:**

A total of 349 patients were included in the study. They completed a survey about their demographic characteristics, psycho-socio-economic data, and disease-related information. Their disability level was assessed using the Expanded Disability Status Scale (EDSS) based on the neurological examination performed by the same doctor for every patient and the SC were documented with the Beck Depression Inventory-II questionnaire.

**Results:**

The study included 112 men and 237 women, with a mean age around 42 years old. Suicidal thoughts were more frequent in men, while suicidal intentions in women. Positive correlations were found between SC and depression, EDSS, total number of relapses, disease duration, and level of education. From the EDSS functional scores, only the pyramidal score and the cerebellar score presented a significant correlation with SC. None of the patients with clinically isolated syndrome had SC. The type of disease-modifying therapy, marital and occupational status, and the presence of children did not influence the presence of SC.

**Conclusions:**

The prevalence of SC is higher in patients with MS compared to the general population. Their occurrence is mostly influenced by the disease itself (duration, relapses, acquired disability) and also by depression and lack of education.

## 1. Introduction

Multiple sclerosis (MS) is known as one of the most common disabling conditions among young people. Although the most striking symptoms appear to be the ones that cause the physical disability (such as visual loss, motor deficits, sensitive, and cerebellar symptoms), the psychological and psychiatric manifestations of the disease (such as depression, behavioral changes), that can sometimes act as “silent killers” leading to suicidal ideation, have come to the attention of researchers over the last several years. Even though Charcot was the first (in the nineteenth century) who described MS's psychiatric manifestations in his lectures at Salpetriere Hospital, these aspects were only more recently debated, playing an important part of the quality of life in MS patients. These symptoms can be easily missed at a routine follow-up visit, especially if the patient has good coping mechanisms [1, 2].

The most frequently reported psychiatric symptoms are depression, dysphoria, agitation, anxiety, and irritability [3, 4]. In the development of depression in the MS patients, a crucial factor, with respect to the psychological aspects of the disease, is the strain of being diagnosed with an incurable, progressive, and unpredictable condition [5]. Although MS is defined as a progressive, neurodegenerative disorder, according to Rolak, the life expectancy is reduced only by a few months and after 15 years from the onset only about 20% of patients will be restricted to their beds [6]. However, not every patient is willing to accept these statistics. Depression in MS is not only a frequent feature of the clinical picture but is also among the most important determinants for suicidal intent and completion [7, 8].

Suicide has always been a tragic, emotional, debatable, and controversial subject from many points of view. Suicide is defined as the act of deliberately ending one's life. Suicidal behavior, which can be explained as an individual's elaborated set of strategies, ideas, and actions designed to commit suicide, is classified in an escalating order into suicidal ideation, plan, and attempt. Suicidal behavior is far more common (10-20 times) than completed suicide, but it remains officially underreported due to social, legal, and economic implications [9, 10].

According to World Health Organization's data from 2015, one person dies every 40 seconds by committing suicide, this being the second leading cause of death in the 15-29-year-old age group [11]. Suicide attempt is more frequent in women, unmarried people, and patients diagnosed with a psychiatric disorder, whereas suicide completion is more common in men [12]. Regarding suicide in MS patients, it was shown that the risk is twofold higher than that in the general population and is particularly increased for men receiving this diagnosis and within the first year of diagnosis [13].

The aim of the present study was to assess the prevalence and the determinants for suicidal concerns (SC) in a large MS patient group.

## 2. Methods

### 2.1. Patients

We performed an observational, prospective, and cross-sectional study. Three hundred forty-nine patients, who consecutively attended our Regional MS Centre over a six-month period, were included in this study. Inclusion criteria consisted of several parameters: (1) age over 18 years; (2) diagnosis of clinically isolated syndrome (CIS), relapsing-remitting MS (RRMS), and/or secondary progressive MS (SPMS) according to McDonald criteria 2010 [14]; (3) undergoing treatment with interferon (IFN) beta-1a and beta-1b, glatiramer acetate, and natalizumab (these were the only disease-modifying therapies available in Romania at the time of the study); and (4) signed informed consent to participate in the study. The exclusion included several parameters: (1) recurrence with corticosteroid treatment in the last month; (2) diagnosis of depression or concomitant treatment with antidepressants; (3) participated in clinical trials on experimental therapy; (4) diagnosed with other chronic diseases (neoplasia, epilepsy); and (5) surgery or hospitalization in the last month. All of the patients completed a survey about their demographic characteristics, psycho-socio-economic data (marital and occupational status, presence of children, education), and disease-related information (disease and disease-modifying therapy (DMT) duration, type of DMT, MS course, number of relapses). Their disability levels were evaluated using the Expanded Disability Status Scale (EDSS), which was obtained by the same neurologist for all patients [15]. SC were documented with questions from Beck Depression Inventory-II (BDI-II), the Romanian version from 2012 [16]. The patients who reported that they had been thinking about committing suicide in the last 2 weeks, but would not do so (score 1), were considered to have suicidal thoughts (ST) and those who reported that had intended to commit suicide (score 2) or that would take life if given the chance (score 3) were considered to have suicidal intentions (SI). The study was conducted in accordance with the principles set out in the Helsinki Declaration. All patients signed the informed consent.

### 2.2. Statistical Analysis

We used the Statistical Package for Social Sciences for all statistical calculations [17]. The Shapiro-Wilk test was used to assess the normality of continuous variables. The difference between means of continuous variables was assessed using Student's *t*-test (expressed as the mean ± standard deviation), while differences between nonparametric variables (express as median, range) were compared with the Mann–Whitney test. The Kruskal-Wallis test was used to estimate the difference among the other variables, which is appropriate for more than two groups. Associations between categorical variables were assessed using contingency tables and chi-squared test. Correlations between quantitative variables were analyzed with Spearman's rho. All tests were interpreted against a *p* = 0.05 significance threshold; statistical significance was considered for values below this threshold.

## 3. Results

The demographic-, psycho-socio-economical-, and MS-related data are presented in Table 1.

From the entire group of MS patients, 314 (89.97%) patients reported not thinking to take their life (score 0), 17 (4.87%) were thinking about committing suicide but would not do so (score 1), 15 (4.29%) intended to commit suicide (score 2), and 3 (0.87%) would take their own life if given the chance (score 3).

The study included 112 men and 237 women. We observed no statistically significant differences regarding the presence of suicidal ideas and intents between the two sexes. ST were found in 3.8% of women and 7.1% of men, while SI were observed in 5.9% of women and only 2.7% in men.

**Figure 1:**
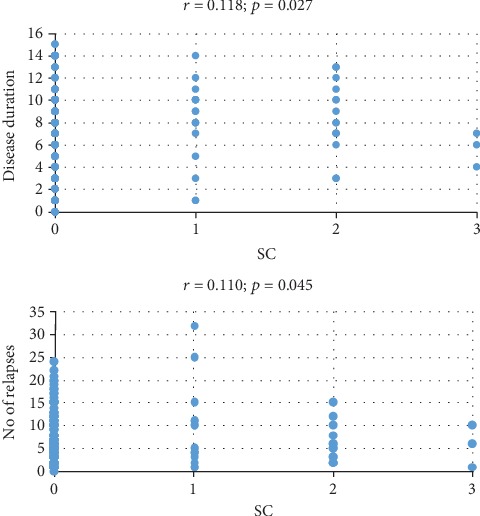
Correlation between SC and disease duration and between the number of relapses and SC.

Using univariate analysis and a Spearman correlation, we obtained positive correlations between SC and disease duration (*p* = 0.027, *r* = 0.118) and the number of relapses, respectively (*p* = 0.045, *r* = 0.110) (Figure 1).

**Figure 2:**
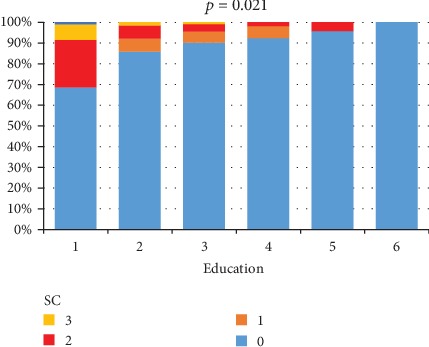
The relationship between SC and education level: 1—elementary school, 2—apprentice school, 3—high school, 4—university, 5—master or PhD, and 6—technical and vocational high school.

The influence that education had on SC is presented in Figure 2. Most patients with a university degree (92.3%) and 95.7% of the postgraduates had a score of 0, while this score was found in only 69.5% of those with secondary school studies. SI were found in 30.8% of patients with secondary education and only in 1.9% of those with university education. The differences were statistically significant, *p* = 0.021.

**Figure 3:**
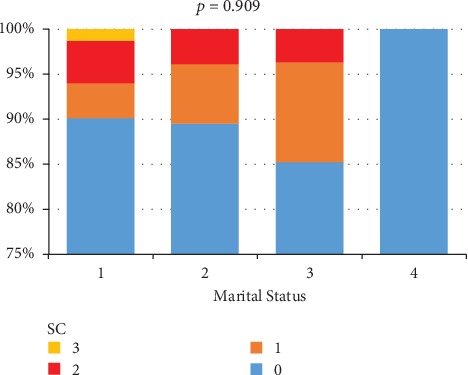
The relationship between SC and marital status: 1—married/living with partner, 2—single, 3—divorced, and 4—widow.

Occupational status did not significantly influence the presence of suicidal ideas (*p* = 0.998). No statistically significant differences regarding the presence or absence of suicidal ideas were registered among the four patient groups, that were divided according to marital status (*p* = 0.909). We believe, however, that it is important to note that of the 18 patients with SI, 77% were married, 16.6% were unmarried, and 5.5% were divorced (Figure 3).

**Figure 4:**
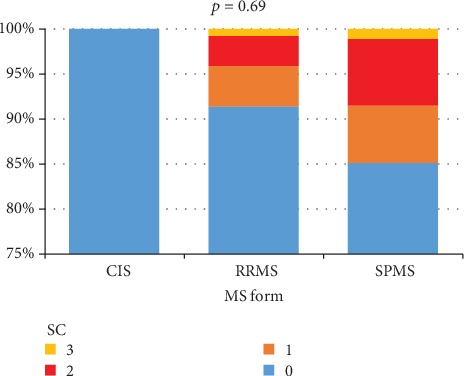
The relationship between SC and MS form.

Concerning the clinical form of MS, 100% of CIS patients had a score of 0. SI were traced to 4.1% of patients with RRMS and 8.5% of those with SPMS. The difference was not statistically significant (*p* = 0.69) (Figure 4).

**Figure 5:**
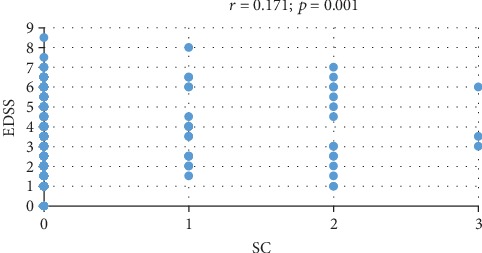
Correlation between the EDSS and SC.

SC presence and severity was positively correlated with the EDSS score (*p* = 0.001, *r* = 0.171) (Figure 5). Of the eight functional scores, SC only correlated with the pyramidal (PFS) and cerebellar (CFS) ones (*p* = 0.02, *p* = 0.005). We found no SI nor ST for 92.7% of the patients with a PFS of 0 and only for 50% of those with a PFS of 5. SI were observed in 3.6% of the patients with PFS 0 and 14% of those with PFS 4. Most patients with CFS 0 (92.4%) had no suicidal ideas or SI, and only 66.7% of those with a score of 4 were in the same situation. SI were present in 3.5% of patients with CFS 0 and 33.3% of those with CFS 4.

The type of DMT, MS course, marital and occupational statuses, and presence of children did not influence the prevalence of SI.

**Figure 6:**
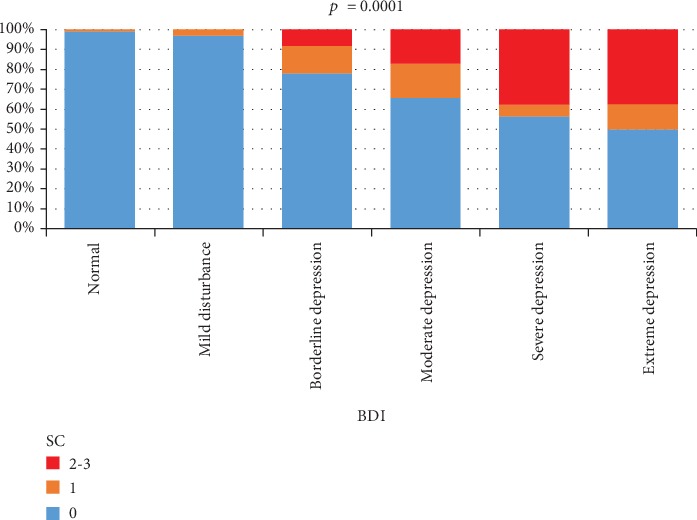
The relationship between SC and BDI score. Normal: <10 points, mild disturbance: 11-16 points, borderline depression: 17-20 points, moderate depression: 21-30 points, severe depression: 31-40 points, and extreme depression: >40 points.

SC presence and severity was strongly correlated with the total score obtained from the BDI-II questionnaire (*p* = 0.0001). The majority of patients with BDI-II score < 10 (98.9%) have not had SI or ST, and only 54.2% of those with BDI-II score ≥ 31 were included in the same category. SI were observed in 0% of those without mood disorders and with mild disturbances, in 8.3% of those with borderline depression, 17.2% of those with moderate depression, and 37.5% of those with severe depression (Figure 6).

## 4. Discussion

Over the past few years, the issue of psychiatric manifestations and especially the suicide matter in MS patients had attracted the attention of many researchers.

It has been established by the epidemiological data that the suicide rate in these patients is higher than that in the general population. Most of the information was retrieved from the Northern European countries thanks to their extensive databases.

The standardized mortality ratio (SMR) was the most used method to quantify the suicide rate. SMR represents the number of deaths in people with MS in a given period divided by the number of deaths expected in the general population on the same loop of time. A value > 1.0 shows an increased suicide rate with or without statistical significance [18].

Different time-lapse studies based on large numbered population cohorts (ranging from 1595 to 12834 people) returned significant SMR values as it follows: 2.3 (from a Swedish research) [13], 1.62, 1.83, and 2.12 from three Danish studies [19–21] and 1.7 from a Finnish study [22]. Other investigators used a different tool to estimate the completed suicide rate by returning a hazard value (which is an estimation from a Cox regression analysis also adjusted for demographics and timeline) of 1.87 (95% confidence interval: 1.53-2/3) similar to the ones described above [23].

The present study also is aimed at drawing the attention towards the psychiatric aspects of MS, quantifying the prevalence of SC and searching for their determinants in a large MS patient group.

The prevalence of suicidal behavior data presents fluctuations in the literature. Its variability could be explained by the heterogeneity of the studied groups and also by the methods used for interviewing the patients.

Three studies have applied the Patient Health Questionnaire which includes nine questions such as “Little interest or pleasure in doing things?” or “Thoughts that you would be better off dead or of hurting yourself in some way?”. The subject answers each item from “not at all” to “nearly every day” adapting it to the events over the last two weeks. Thus, these thoughts are assessed as suicidal or self-harmful thoughts. Viner et al. studied 188 patients with MS. At baseline, 8.3% of them endorsed ST, but this value increased to 22.1% over the course of six months [24]. Turner et al. also applied the Patient Health Questionnaire to 445 MS patients from the Veteran's Health Administration and obtained a surprisingly high prevalence of suicidal ideas (29.4%) and persistent ST (35%) [25]. In a larger group (3823 patients), Dickstein et al. found a suicidal behavior prevalence of 15% [26].

In another study conducted by Feinstein, 140 MS subjects were questioned using the Beck Suicide Scale and the Structured Clinical Interview for DSM IV Axis 1 Disorders, and 28.6% of participants had lifetime SI [7]. A German study concerning 867 people revealed that 22.1% of them answered “often,” “very often,” or “all the time” when asked “How often do you think about ending your life?” [27].

Lee et al. conducted a study to evaluate the prevalence of suicidal ideation in the general population. There were included 2054 people in the study, who were interviewed by telephone and 2.84% reported suicidal ideation in the last week [28].

The prevalence of SC in our group of patients was 10.03%, the value that is the most consistent with the published data (11.76%) as revealed in Ben-Zacharia's research who also used BDI-II [29]. So, it seems like 10 in 100 patients with MS would consider taking their lives even though they have not been previously diagnosed with any mental or other chronic illness that could increase their suicide risk.

In their review from ten years ago, Nock et al. described suicide and suicidal behavior in the United States and cross-nationally and showed that, in the general population, the rate of suicide is higher in males than in females. This pattern, despite the broad fluctuation, is characteristic for both the United States and other countries. Men die from suicide at a ratio ranging from 3 : 1 to 7.5 : 1. On the other hand, when it comes to suicidal behavior, the study revealed opposing results because the rate of nonfatal self-injury was more common in females [10]. Starting from the premise that men are more predisposed to complete suicide while women more often attempt suicide, we could expect that this behavior could be maintained in the MS population because of the progressive nature of the disease and the idea of being diagnosed with an incurable condition. However, this has not been proven as a rule. Some studies have shown that males with MS were more likely to have a suicidal ideation (odds ratio 1.52, *p* < 0.001) than females [26], while others revealed high frequencies in both men and women for attempting suicide and suicide risk as SMR with no statistical significance [13, 23].

Fredrikson et al. revealed though a higher suicide risk in the first year of MS diagnosis and among younger males. He also found a suicide rate of 71 per every 100,000 persons/year among MS patients with a statistically significant difference between men and women (*p* < 0.001) [13].

Bronnum-Hansen et al. studied suicide among Danes with MS based on the Danish Multiple Sclerosis Registry updating a previous study concerning the same subject from 1992. They wanted to establish if the suicide risk among MS patients has changed since the mid-1900s. The SMR was overall similar for men and women (2.16 versus 2.07). After 15 years from diagnosis, the excess suicide rate has changed only for women from 4.03 to 1.73 in the subsequent periods, while it remained nearly steady for men. The risk appears to have decreased in the 15 to 20 years after the diagnosis and increased again after 20 years with a slightly higher risk for women (2.04 compared to 1.74). However, the calculated SMR had no statistically significant difference when comparing the two sexes or the variations over a time lapse in the same sex [21].

Our study included 112 men and 237 women. ST were found in 3.8% of women and 7.1% men, while SI have an opposite distribution with 5.9% in women and only 2.7% in men. However, no statistically significant differences between the two sexes regarding SI and ST were observed. Our results are consistent with those from the study performed by Turner and his colleagues who also did not return any statistical significance in a bivariate relation between suicidal ideas and gender [25].

Being diagnosed with a chronic condition such as MS is a burden, aside from the disabling character of the disease itself. Suicide in these patients could be seen as “being defeated by the long hard struggle to stay alive,” as Brampton said in his book [30].

The World Health Organization defines disability as an “umbrella term” for a complex phenomenon that reflects the interactions between one's body functions and features of the society in which the person lives. It covers impairment (a problem in body function or structure), activity limitations (a difficulty in executing a task or action), and participation restrictions (a problem experienced when an individual becomes involved in life situations) [31].

Literature reports indicate that different types of disability such as limited activity, perceived and actual disabilities, and physical limitations are associated with suicidal ideation [32, 33].

Disability in MS is caused by the accumulation of different neurological impairments such as motor, sensory, coordination, gait, and visual symptoms as the disease concurrently affects distinct parts of the white and grey matter in the central nervous system. Aside from these manifestations, there is also a series of “hidden disabilities” that include difficulties with cognition, memory, mood, affect, pain, fatigue, sleep, bowel, bladder, and sexual functions that could be easily missed in a routine consultation [34]. Quantifying the disabilities in MS helps us to assess the disease progression, monitor clinical outcomes in individual patients for better care, and also evaluate the treatments' effects. The most universally used tool for measuring the disability and progression in MS is the EDSS developed by Kurtzke in 1983 [15].

After searching the literature for a direct relationship between SI and disability, more studies seem to lean towards a positive correlation. However, a large-scale study performed by Feinstein did not find a significant association [7].

Gaskill et al. performed a qualitative study in which they found that frustrations related to limited functioning and perceived loss of control were among the eight key themes identified in people with MS experiencing suicidal ideas, the last mentioned being the most common one [35].

Two more studies described that communication and swallowing difficulties and self-reported bowel and bladder problems were risk factors for SI [24, 25].

Other studies reported that moderate and severe levels of disability are risk factors for completed suicide in MS [36, 37].

A few studies revealed that the progressive MS types appear to be associated with higher levels of suicidal ideas because of the increased level of disability which characterize them, when compared with RRMS form [25, 38–40].

Regarding the disease duration, Bronnum-Hansen et al. found that SMR was higher for persons diagnosed for >20 years [21].

We also used the EDSS for our study group, and it was based on the neurological examination performed by the same doctor for all the study patients. We obtained a positive correlation between the presence and the severity of SC with the EDSS. Statistically significant values were found also when we correlated SC with PFS and with CFS, respectively. This draws the attention towards the fact that for our patients, losing control of the motor functions and not being able to coordinate one's movements are the most disabling situations that can lead to suicidal behavior. Although we did not find a positive correlation regarding the autonomic functions on EDSS and SC, it is important that almost 95% of the patients with a bowel and bladder functional system of 0 had no SI or ST.

Relapses also add neurological impairments to the baseline of the functional status, even for a limited period of time, and we were not surprised that we found positive correlations between the number of relapses and SC. Also, the disease duration correlated positively with SI and ST which was consistent with the results of Bronnum-Hansen stated above [21].

Although the clinical forms of MS did not correlate with SC, it is worth mentioning that 100% of CIS patients had no SC, and more patients with SPMS (8.5%) had SI compared to those with RRMS (4.1%), the last finding being similar with the literature [25, 38–40].

Two studies conducted by Cerqueira and Brenner found correlations regarding the impact of education on the SC which agree partially with our results. Cerqueira et al. compared two groups of MS patients: one at risk of suicide and one without it (the control group) and they found that for the control group the education level was higher. Brenner et al. observed that the risk for attempting suicide was lower for both MS and non-MS patients with higher education levels, but the association for completed suicide was seen only in the non-MS group [23, 41].

On this matter, we found that 92.3% of patients with a university degree and 95.7% of the postgraduates had no ST, and SI were observed only in 1.9% of those with a university education, in contrast to 30.8% of patients with secondary education. This difference was statistically significant, so it seems that the lower the education level, the higher the incidence of SI. This could mean that a higher education level may give those patients a different perspective over the idea of living with MS. They could be more easily approached about the importance of treatment, the clinical and imaging follow-ups, and the possibilities of dealing with their disability. They may also have good coping mechanisms that help them gain confidence over the fact that life does not end with this diagnosis.

Of the DMT's used in our study, IFN beta raise the question whether there is a link between this therapy and suicide. In the literature, the relationship between IFN beta therapy and suicidal behavior is unclear. The first IFN beta-1b trial concerning RRMS patients raised the suspicion as to whether this treatment could be linked to suicide because completed suicide and suicide attempt were documented for the treatment arms but not for the placebo group [42]. Additional studies have documented the association between IFN beta and the presence of symptoms for severe depression [43], and Goeb and his colleagues concluded in their review of IFN beta-1a and beta-1b that treatment itself has no greater risk of suicidal behavior [44]. However, in clinical practice, all patients undergoing treatment should be closely monitored for signs of suicidal thinking [18].

We did not see an influence of the DMT type on the prevalence of SC, but we found that 70.6% of the patients with ST and 66.7% of those with SI were treated with different types of IFN beta.

Social support is an important component in the psychological balance of each individual and even more so for a person suffering from various medical conditions. Lower social support levels are associated with an increased in vulnerability to stressors and a tendency towards physical and psychological health problems. Furthermore, people with suicidal behavior described less support from their friends and family [45, 46].

Arciniegas and Anderson stipulated that social isolation represents an increased risk factor for suicide in patients with neurological illnesses [47].

This finding was also reported in the case of MS. In the studies performed by Feinstein and Bronnum-Hansen and his colleagues, it was found that social isolation can be among the predictors for suicidal ideation in MS patients [7, 21].

In the study performed by Turner and his collaborators, lower levels of perceived social support were associated with suicidal ideas. He also found for the first time that lower income is a variable of the same equation. However, no relationship between living alone and suicidal behavior was noticed [25].

Long and Miller suggested that suicidal tendencies in MS patients could be predicted using the concept of family support [48].

Social support from family, friends, and significant others was found to be negatively correlated with SI in MS patients in a relatively recent study by Ariapooran and his team [49].

In another study conducted by Cerqueira et al., it was found that with respect to marital status, the risk of suicide was higher for single, separated, or widowed individuals when compared with the control group [41].

Although in our study group, occupational or marital status did not significantly influence the presence of SC, it is important to notice that of the 18 patients who express SI, 77% were married/living with a partner, 16.6% were unmarried, and 5.5% were divorced. It seems that for the Romanian population having a diagnosis of a chronic disease like MS carries the biggest chance of developing SI when a person is married or living with a partner. This does not mean that our patients have little or no family support, but from their points of view, they may see themselves as a burden for their relatives. However, this should not lead us to draw any hasty conclusions and the problem should be studied within an extended framework of what social support means, not just the one offered by the family environment.

It is well known that mental illnesses carry a great risk for patients for harming or killing themselves and depression in all stages is no exception. The risk of suicidal ideas and attempts for patients suffering from mood disorders was estimated at 51% and 44%, respectively, and 60% of completed suicide cases had a history of depressive episodes [50, 51].

Among all mental health conditions, depression is the most common one in the general population but is also an important and frequent comorbidity in other chronic pathologies such as neurodegenerative diseases and MS [12, 52, 53].

In the general population, the prevalence of depression varies from 20.6% in South America to 7.3% in Australia [52], but when it comes to MS, the prevalence is two- to threefold higher, ranging from 40% to 60% making depression the most frequent psychiatric manifestation of MS. In addition to this finding, people with MS suffer from more severe depressive symptoms hence the frequent association between depression, suicide, and MS [54–56].

The symptoms of depression in MS occur secondary to an interaction between the basic psychological structure of an individual and his reaction to the diagnosis, together with the changes following neurodegeneration, possible side effects of the medication, and the social support levels [43, 57–59].

The suicidal ideation is, in fact, a core symptom for major depression. In other words, referring to depression in MS, we could state that it is one of the strongest risk factors for suicidal ideation [60, 61].

This association between depression and suicide was found by Cerqueira et al. in their study in which they also observed the presence of other psychiatric disorders such as bipolar disorder, psychotic syndrome, abuse and dependence on drugs, and bulimia nervosa in the suicide risk group [41].

Other researchers such as Viner, Turner, Fisk, and their colleagues endorsed the same correlation [24, 25, 62]. Fisk et al. studied the prevalence of depression, bipolar disorder, and attempted suicide among patients who used hospital psychiatric resources and compared those characteristics between the MS and non-MS groups. They concluded that bipolar disorders and depression on one hand and the frequency of suicide attempts on the other were two and, respectively, three times greater in the MS patients group [62]. Turner et al. found that suicidal ideas were significantly more common among patients with a higher depression severity score [25].

It has been found that depression not only is a risk factor itself for suicidal ideation but also acts as a mediator among other determinants of suicidal behavior. In their study, Lewis and his colleagues observed that depressive symptoms had an indirect effect on the relationship between actual and perceived disability and suicide ideation in MS [33].

In our study, we found the strongest correlation between the presence and severity of SC and the total score of the BDI-II questionnaire (*p* = 0.0001). No SC were found for 98.9% of those with a score < 10. SI were present in 37.5% of those with severe depression, while those suffering from mild disturbances or those without any mood disorders had no SI. These results are consistent with preexisting studies mentioned before and perpetuate the idea of suicide being the most dramatic facet of major depression.

To improve a further research, we could extend our statistics not only for suicidal ideation but also for suicide attempts and completed suicide at a national level, apply more tests or questionnaires concerning suicidal behavior, searching for persistent suicidal ideation over a longer time period, and after treating the subsequent depressive symptoms. We could also study if any other concomitant psychiatric disease influences the suicidal behavior or if MS has an effect on a preexisting psychiatric pathology regarding the suicidal behavior.

## 5. Conclusions

The prevalence of SC is higher in patients with MS compared to the general population. The main determinants for SC that we found are depression, an increased level of disability, a high number of recurrences, a longer duration of illness, and a low level of education.

Suicide is a tragedy, but we have the tools to prevent it. Searching for the determinants of suicidal ideation should now be a part of the routine examination. Among all of them, depression is the most common and highly associated with SC. A big step forward in avoiding suicide among MS patients is to suspect and diagnose depression in an early stage because, unlike other facets of MS, it is the most receptive to treatment.

## Figures and Tables

**Table 1:** Demographic-, psycho-socio-economical-, and MS-related data.

	Without ST and SI 0	With ST 1	With SI 2-3
Gender			
Female	213-67.9%	9-52.9%	15-83.3%
Male	101-32.2%	8-47.1%	3-16.7%
Age (mean/SD)	42.85/9.75	42.59/8.91	45.5/8.71
Disease duration			
Median (min–max)	10 (0-39)	10 (2-28)	11 (7-34)
Type of MS			
CIS	10-3.2%	0-0.0%	0-0.0%
RRMS	224-71.3%	11-64.7%	10-55.6%
SPMS	80-25.5%	6-35.3%	8-44.4%
Treatment duration			
Median (min–max)	7 (0-15)	9 (1-14)	7.5 (3-13)
Type of treatment			
AVONEX	44-14%	3-17.6%	0-0.0%
BETAFERON	121-38.5%	7-41.2%	9-50%
COPAXONE	58-18.5%	2-11.8%	6-33.3%
REBIF	78-24.8%	2-11.8%	3-16.7%
TYSABRI	13-4.1%	3-17.6%	0-0.0%
EDSS			
Median (min–max)	2.5 (0-8.5)	4 (1.5-8)	3.25 (1-7)
Number of relapses			
Median (min–max)	4 (0-24)	5 (1-32)	5.5 (1-15)
Marital status			
Married/living with partner	210-67.1%	9-52.9%	14-77.8%
Single	68-21.7%	5-29.4%	3-16.7%
Divorced	23-7.3%	3-17.6%	1-5.6%
Widow	12-3.8%	0-0.0%	0-0.0%
Educational level			
Elementary school	9-2.9%	0-0.0%	4-22.2%
Apprentice school	54-17.3%	4-23.5%	5-27.8%
High school	119-38%	7-41.2%	6-33.3%
University	96-30.7%	6-35.3%	2-11.1%
Master or PhD	22-7.0%	0-0.0%	1-5.6%
Technical and vocational high school	13-4.2%	0-0.0%	0-0.0%
Occupational status			
Employed	125-39.9%	6-35.3%	4-22.2%
Part-time employed	20-6.4%	1-5.9%	1-5.6%
Disability pension	140-44.7%	10-58.8%	13-72.2%
Retired	8-2.6%	0-0.0%	0-0.0%
Student	2-0.6	0-0.0%	0-0.0%
Unemployed	17-5.4%	0-0.0%	0-0.0%

## Data Availability

The data that support the findings of this study are available from the corresponding author, N.Ş., upon reasonable request.

## Abbreviations

BDI-II: Beck depression inventory-II
CFS: Cerebellar functional score
CIS: Clinically isolated syndrome
DMT: Disease-modifying therapy
EDSS: Expanded disability status scale
IFN: Interferon
MS: Multiple sclerosis
PFS: Pyramidal functional score
RRMS: Relapsing-remitting multiple sclerosis
SC: Suicidal concerns
SI: Suicidal intentions
SMR: Standardized mortality ratio
SPMS: Secondary progressive multiple sclerosis
ST: Suicidal thoughts.
